# Antioxidant and Antifungal Activities and Characterization of Phenolic Compounds Using Ultra-High Performance Liquid Chromatography and Mass Spectrometry (UPLC-MS) of Aqueous Extracts and Fractions from *Verbesina sphaerocephala* Stems

**DOI:** 10.3390/plants13192791

**Published:** 2024-10-05

**Authors:** Kathia Yanelly Rodríguez-Valdovinos, Rafael Salgado-Garciglia, Alejandra Hernández-García, Alfredo Saavedra-Molina, Rosa Elva Norma del Río-Torres, Joel Edmundo López-Meza, Juan Luis Monribot-Villanueva, José Antonio Guerrero-Analco, José Roberto Medina-Medrano

**Affiliations:** 1 Instituto de Investigaciones Químico Biológicas, Universidad Michoacana de San Nicolás de Hidalgo, Morelia 58030, Michoacan, Mexico; 2019606d@umich.mx (K.Y.R.-V.); rafael.salgado@umich.mx (R.S.-G.); alejandra.hernandez@umich.mx (A.H.-G.); francisco.saavedra@umich.mx (A.S.-M.); norma.del.rio@umich.mx (R.E.N.d.R.-T.); 2 Centro Multidisciplinario de Estudios en Biotecnología-FMVZ, Universidad Michoacana de San Nicolás de Hidalgo, Tarímbaro 58880, Michoacan, Mexico; elmeza@umich.mx; 3 Red de Estudios Moleculares Avanzados, Clúster BioMimic®, Instituto de Ecología, A.C., Xalapa 91073, Veracruz, Mexico; juan.monribot@inecol.mx (J.L.M.-V.); joseantonio.guerrero@inecol.mx (J.A.G.-A.); 4 Licenciatura en Genómica Alimentaria, Universidad de La Ciénega del Estado de Michoacán de Ocampo, Sahuayo 59103, Michoacan, Mexico

**Keywords:** antifungal activity, antioxidant activity, *Botrytis cinerea*, phenolics, *Verbesina sphaerocephala*

## Abstract

The *Verbesina* gender represents the second most diverse group from the Asteraceae family in Mexico; *Verbesina sphaerocephala* is one of the most distributed species along the Mexican territory. This species has been poorly studied, reporting the presence of some bioactive compounds with antioxidant and antibacterial activity. In this study, phenolic and flavonoid contents and composition, antioxidant and antifungal activities of aqueous extracts of the stem of *V. sphaerocephala* and its fractions were determined. The results showed that the highest antifungal activity against *Botrytis cinerea* was shown by the aqueous extract (IC_50_: 0.10 mg/mL) and the ethyl acetate fraction (IC_50_: 14.8 mg/mL). In addition, the aqueous extract and the ethyl acetate fraction exhibited the highest phenolic (21.40 and 21.26 mg gallic acid equivalents per gram of dry extract, respectively) and flavonoid contents (11.53 and 3.71 mg rutin equivalents, respectively) and high antioxidant activity determined by the Total Antioxidant Capacity (20.62 and 40.21 mg ascorbic acid equivalents per gram of dry extract, respectively), Ferric Reducing Power (74.76 and 129.57 mg gallic acid equivalents per gram of dry extract, respectively), DPPH (IC_50_: 12.38 and 7.36 mg/mL, respectively), and ABTS (IC_50_: 5.60 and 7.76 mg/mL, respectively) methods. Twelve phenolic compounds were detected in the aqueous extract using UPLC-MS analysis, of which the major ones were protocatechuic, vanillic, and hydroxybenzoic acid, while in the ethyl acetate fraction, the presence of 18 phenolic compounds were identified, of which the majority were vanillin, rutin, and hydroxybenzoic acid. The results of this research demonstrate that the aqueous extract of *V. sphaerocephala* stems has phenolic compounds with antifungal and antioxidant activity.

## 1. Introduction

Fungi are one of the phytopathogens that mostly attack plants and cause diseases, both in the pre- and post-harvest stages [1]. It is reported that there are more than 19,000 fungi that cause disease in plants in the world [2]. *Botrytis cinerea* is a necrotrophic fungus that causes serious economic and agronomic losses worldwide [3]. This fungus affects nearly 500 crops worldwide, the main ones being grapes, berries (strawberries, blackberries, blueberries, raspberries), Solanaceae (tomatoes, potatoes), and pumpkins, among others [4]. The fight against *B. cinerea* and other phytopathogenic fungi has driven research into new control strategies, with a growing focus on the use of natural compounds as sustainable alternatives to conventional chemical treatments.

Phenolic compounds, known for their antioxidant and antimicrobial properties, have a high potential to be used in the inhibition of the growth of phytopathogenic fungi. In addition, they are considered low toxicity since they have a short persistence in the environment. These plant metabolites are potentially considered to be integrated as one of the alternative methods for the control of phytopathogenic fungi [5,6,7].

*Verbesina sphaerocephala* is an endemic plant species of Mexico [8], belonging to the Asteraceae family, known for its medicinal properties and its wide distribution in tropical and subtropical regions. In recent years, this plant has attracted the attention of researchers due to its therapeutic potential, particularly with regard to its antioxidant, antibacterial, and antifungal properties and its phenolic composition. The antioxidant activity of *V. sphaerocephala* has been documented in several studies, which highlight its ability to neutralize free radicals and reduce oxidative stress. This property is attributed in part to its rich phenolic composition, which includes compounds such as flavonoids, phenols, and phenolic acids, known for their ability to protect cells against oxidative damage and premature aging [9,10,11]. In addition to its antioxidant activity, *V. sphaerocephala* has shown promising antibacterial properties. Recent research has shown its effectiveness against pathogenic bacteria such as *Staphylococcus aureus* and *Escherichia coli*, suggesting its potential use in the treatment of bacterial infections [11]. Regarding its antifungal activity, the plant has been shown to be effective against various pathogenic fungi, including *Colletotrichum gloeosporioides*, *Fusarium oxysporum*, and *Botrytis cinerea* species, which opens the possibility of its use in the treatment of fungal infections [12]. These findings underline the importance of *V. sphaerocephala* as a potential source of bioactive compounds that can be used in the development of new agents for the management of *B. cinerea*.

Therefore, the aim of the present study was to determine the phenolic and flavonoid contents, phenolic composition, and in vitro antioxidant and antifungal activities against *B. cinerea* of aqueous extracts and its fractions from *V. sphaerocephala* stem.

## 2. Results and Discussion

### 2.1. Total Phenolic and Flavonoid Content

In this research, the phenolic and total flavonoid content of the aqueous extract and its fractions from the stem tissue of *V. sphaerocephala* were evaluated. Table 1 shows these results. The range of phenolic content was from 21.40 to 13.54 milligrams equivalent of gallic acid per gram of dry extract (mg GAE/g DE), with the aqueous extract having the highest content (Table 1). In previous research on this plant, a total phenolic content of 10.50 mg GAE per gram of dry tissue (DT) was reported in methanolic extracts of the leaf tissue [11], two times lower than what was obtained in this study.

In the case of the aqueous extract fractions obtained by liquid–liquid phase separation, the ethyl acetate fraction was the one that showed the highest content (21.26 mg GAE/g DE), while the methanolic fraction obtained the lowest content (13.54 mg GAE/g DE), as can be seen in Table 1. There are no previous studies on the fractionation of extracts in this plant, so it is proposed to carry out more work in this area.

On the other hand, in the total flavonoid content of the extracts and fractions, a similar behavior to that of the phenolic content results was observed since the highest flavonoid content was presented in the aqueous extract with 11.53 milligrams of rutin equivalents per gram of dry extract (mg RE/g DE). The flavonoid content of the aqueous extract analyzed in this study was higher compared to that obtained in the hydro-methanolic extract of leaves of this plant previously reported [11], which was 9.85 mg equivalents of catechin per gram of dry tissue (mg CE/g DT), being 1.17 times higher than that obtained in the aqueous extract of stems reported here. Meanwhile, the ethyl acetate fraction of the aqueous extract showed the highest flavonoid content with 3.71 mg RE/g DE, while the methanolic fraction showed a content of 2.12 mg RE/g DE, which was the lowest flavonoid content.

### 2.2. Identification and Quantification of Phenolic Compounds by Ultra-High-Performance Liquid Chromatography Coupled to Mass Spectrometry (UPLC–MS)

According to the UPLC–MS analysis carried out on the aqueous stem extract from *V. sphaerocephala* and its fractions (ethyl acetate and methanol), the presence of 12 phenolic compounds was found in the aqueous extract, 18 compounds in the ethyl acetate fraction and 13 compounds in the methanolic fraction (Table 2). In the aqueous extract, 84.61% of these compounds correspond to the group of phenolic acids, while the remaining (15.38%) were flavonoids. The major compounds in this extract were protocatechuic acid (96.06 µg/g DE), vanillic acid (66.86 µg/g DE), and hydroxybenzoic acid (65.59 µg/g DE).

On the other hand, in the case of the ethyl acetate fraction, the proportion of its phenolic compounds was the following: phenolic acids (63.15%), flavonoids (26.31%), coumarins (5.26%) and lignans (5.26%). Vanillin (289.27 µg/g DE), rutin (250.52 µg/g DE), and protocatechuic acid (250.54 µg/g DE) were the compounds with the greatest presence. The presence of phenolic compounds with high antioxidant activity, such as isoquercitrin (quercetin-3-*O*-glucoside) [13], astragaline (kaempferol 3-*O*-glucoside) [14], secoisolariciresinol [15] and kaempferol [16] were detected by chromatographic analysis only in the ethyl acetate fraction.

Similarly, in the methanolic fraction, 76.92% of the identified phenolics are phenolic acids, and 23.07% are flavonoids. In this fraction, the majority of compounds were chlorogenic acid (64.05 µg/g DE), protocatechuic acid (16.14 µg/g DE), and rutin (10.28 µg/g DE). Rodríguez-Valdovinos et al. [11] previously detected the compound rutin in *V. sphaerocephala* leaf tissue (13.97 mg/g DT) using High–Performance Thin Layer Chromatography (HPTLC), suggesting that this compound is stored in greater quantities in the leaves and flowers of this plant. Nevertheless, in *Verbesina encelioides*, the presence of *p*-coumaric acid, vanillic acid, gallic acid, caffeic acid, ferulic acid, and chlorogenic acid has been reported [17], which makes them compounds in common between these two species of the genus.

### 2.3. Antioxidant Activity

#### 2.3.1. DPPH Antioxidant Assay

Table 1 shows the antioxidant activity presented by the aqueous extract and its fractions from the stem tissue of *V. sphaerocephala*. As can be seen in Table 1, the ethyl acetate fraction of the aqueous stem extract was the one that presented the highest scavenging activity of the DPPH radical since a concentration of 7.36 mg/mL of extract was required to inhibit the radical, followed by the methanolic fraction and the aqueous stem extract, with IC_50_ values of 8.48 and 12.36 mg/mL, respectively. In previous studies, it has been reported that the methanolic extract of *V. sphaerocephala* leaves showed an IC_50_ value of 5.83 mg/mL [11].

Based on correlation analysis, no clear relationship was observed between the total phenolic and flavonoid contents of the extracts and the scavenging activity of radical DPPH since the extract and fractions that showed the highest total phenolic and flavonoid contents did not obtain the highest DPPH radical scavenging effect.

#### 2.3.2. ABTS Antioxidant Assay

Regarding the scavenging activity of the ABTS radical, the aqueous extract showed the strongest ABTS radical scavenging effect with an IC_50_ value of 5.60 mg/mL. Both the ethyl acetate fraction and the methanolic fraction showed lower scavenging activity of radical ABTS (IC_50_ = 7.76 and 10.26 mg/mL, respectively). These values, however, are lower than those previously reported for methanolic leaf extracts of *V. sphaerocephala* [11].

Based on Pearson’s correlation coefficient, a clear relationship was observed between the ABTS scavenging activity and the phenolic and flavonoid contents (*R* = −0.891, *p* ≤ 0.01 and *R* = −0.918, *p* < 0.01, respectively) of the *V. sphaerocephala* extracts and fractions, which indicates that by increasing the content of phenolics in the extracts and fractions, the scavenging activity increased.

#### 2.3.3. Total Antioxidant Capacity

According to the results of the Total Antioxidant Capacity (TAC) in which the capacity of the extracts to reduce molybdenum (VI) to molybdenum (V) was evaluated, the antioxidant capacity ranged from 20.62 to 40.21 mg AAE/g DE. Similarly, as with the DPPH radical, the ethyl acetate fraction exhibited the highest antioxidant capacity, while the aqueous extract obtained the lowest antioxidant capacity (Table 1). These values are lower than those obtained in methanolic extracts of *V. sphaerocephala* leaves (51.05 ± 0.36 mg AAE/g DT) [11].

#### 2.3.4. Ferric Reducing Power (FRP)

The ferric-reducing power analysis determines the ability of metabolites to reduce the ferric ion (Fe^3+^) to ferrous (Fe^2+^) due to the donation of an electron by measuring the antioxidant activity in equivalents of gallic acid per gram of dry extract. In this work, the ethyl acetate fraction exhibited the highest value of antioxidant activity to reduce ferric ions (129.57 mg GAE/g DE), while the methanolic fraction obtained the lowest value (61.48 mg GAE/g DE). To the best of our knowledge, no reports of this assay have been found for this plant species.

The antioxidant activity of the extract and the fractions showed significant differences (Table 1), as did their phenolic composition (Table 2). Something important to highlight is the increase in the antioxidant activity of the ethyl acetate fraction in all the tests evaluated (DPPH, ABTS, TAC, and FRP) compared to the results with the aqueous stem extract, as shown in Table 1. This event could be due to behavior that occurs in the fractions since the active compounds present in the fractions are less linked to other compounds compared to the extract, in which an antagonism phenomenon could exist, which is why its antioxidant effect could be compromised [18]. A more detailed analysis between the individual phenolic compounds and the DPPH radical scavenging activity showed that chlorogenic acid, which was identified in the aqueous extract and both fractions (Table 2), was one of the compounds that contributed most to the scavenging activity of DPPH radical (*R* = −0.933, *p* < 0.01). In the specific case of quercetin, which was identified only in the ethyl acetate and methanol fractions (Table 2), it could have been responsible for the DPPH radical scavenging activity observed, according to Pearson’s correlation coefficient (*R* = −0.861, *p* < 0.01). The above could explain that both fractions showed higher scavenging activity than the aqueous extract, as can be seen in Table 1. Luteolin, a compound present in the aqueous extract and absent in the fractions (Table 2), may have contributed to the ABTS radical scavenging activity, according to the correlation analysis (*R* = −0.839, *p* < 0.01). Likewise, a positive correlation was observed between 17 of the compounds identified in this research with the ferric reducing power, with *trans*-cinnamic acid (*R* = 0.999, *p* < 0.01), salicylic acid (*R* = 0.998, *p* < 0.01), vanillic acid (*R* = 0.997, *p* < 0.01), *p*-coumaric acid (*R* = 0.996, *p* < 0.01), ferulic acid (*R* = 0.995, *p* < 0.01), protocatechuic acid (*R* = 0.989, *p* < 0.01), rutin (*R* = 0.987, *p* < 0.01), vanillin (*R* = 0.986, *p* < 0.01), having the highest correlation values.

With these results in the four antioxidant tests evaluated, we can determine that the aqueous stem extract of *V. sphaerocephala* and its ethyl acetate fraction have a potential antioxidant effect, so it is necessary to carry out more research to explore new biological properties.

### 2.4. Antifungal Activity

**Figure 1:**
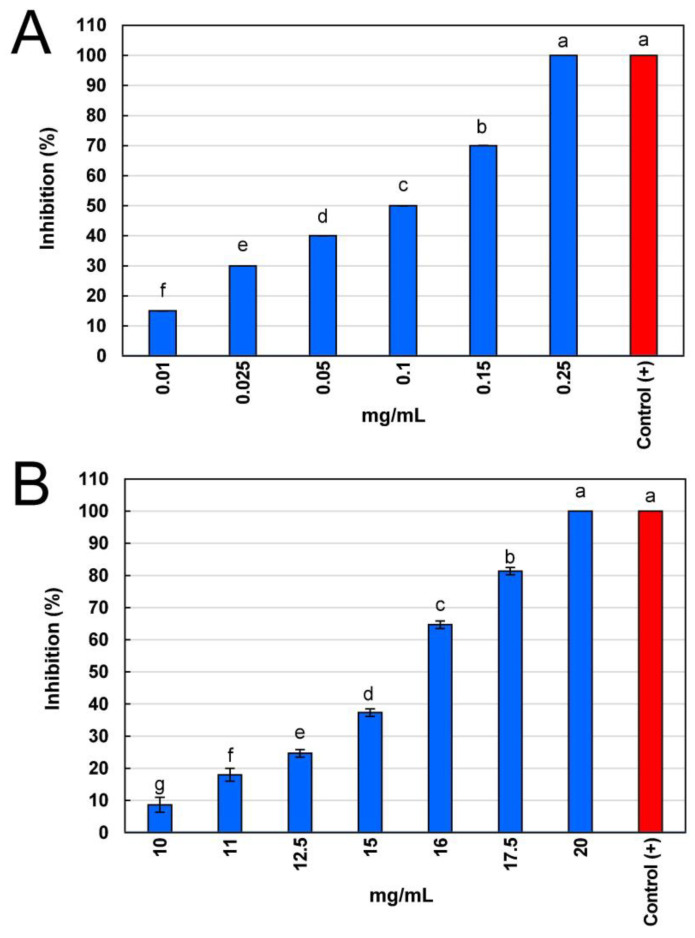
Antifungal activity of stem aqueous extract and ethyl acetate fraction from *Verbesina sphaerocephala* against *Botrytis cinerea*. (**A**) Percentage of inhibition of mycelial growth of aqueous stem extract at different concentrations; (**B**) Percentage of inhibition of mycelial growth of ethyl acetate fraction at different concentrations. Abbreviations: Control (+), Benomyl 10 mg/mL. Values are expressed as mean ± standard deviation of three repetitions. Values with different letters indicate significant differences (Tukey, *p* < 0.05).

Figure 1 shows the results of antifungal activity as the median inhibitory concentration (IC_50_), which represents the concentration of aqueous extract or fraction needed to reduce by 50% (IC_50_) the mycelial growth of *B. cinerea*. Interestingly, the methanolic fraction did not show inhibition against *B*. *cinerea*. The aqueous stem extract showed the highest antifungal activity compared to its ethyl acetate fraction, as their IC_50_ and lethal inhibitory concentration (IC_100_) values were 0.10 ± 0.00 and 0.24 ± 0.00 mg/mL (10 and 24 µg/mL), respectively (Figure 1A). In the case of the ethyl acetate fraction, its antifungal activity was low, showing IC_50_ and IC_100_ values of 14.51 ± 0.06 and 21.49 ± 0.09 mg/mL, respectively (Figure 1B). Interestingly, both the aqueous stem extract and its ethyl acetate fraction showed a fungicidal effect, as there was no mycelial growth after the five days of the assay evaluation.

In a previous study by our working group, we reported the antifungal activity of hydrolates of leaves and stems from *V. sphaerocephala* against the fungi *C. gloeosporioides*, *F. oxysporum*, and *B. cinerea* [12]. In that work, *B. cinerea* was determined to be more sensitive against stem hydrolate with IC_50_ and IC_100_ values of 1.3 and 2.7 mg/mL (130 and 270 µg/mL), respectively. It should be noted that the hydrolates were not as effective as the aqueous extract reported here since up to 13 times more hydrosol was required to inhibit the growth of the fungi *B. cinerea* by 50%.

On the other hand, other species of the genus *Verbesina* have reported antifungal activity against other phytopathogenic fungi, such as the case of *Verbesina lanata*, whose ethyl acetate extract showed an IC_50_ value of 35 μg/mL against the fungus *Plasmopara viticola* [19]. Similarly, other authors report the antifungal activity of *V. encelioides* extracts against *Sclerotium rolfsii*, *Aspergillus flavus*, *Aspergillus niger*, and *Rhizoctonia solani* [20].

As previously indicated, the phenolic composition of the stem extract and fractions of *V. sphaerocephala* varied significantly (Table 2). Different antifungal modes of action have been reported for some of the phenolic compounds identified in both the aqueous extract and its fractions (ethyl acetate and methanolic) of *V. sphaerocephala* stems. Protocatechuic acid acts by affecting membrane permeability as well as cell wall degradation in *B. cinerea* [21]. Likewise, vanillic acid decreases membrane permeability [22] as well as inhibits ergosterol synthesis [23]. Vanillin has different antifungal modes of action, such as increased membrane permeability, leakage of intracellular contents, and inhibition of cell wall enzymes such as polygalacturonase, pectin lyase, and endo-1,4-β-D-glucanase [24]. *p*-coumaric acid alters oxidative phosphorylation, causing uncoupling in mitochondria [25]. Scopoletin acts by inhibiting conidial germination, affecting the high osmolarity glycerol pathway, as well as altering the cell membrane and cell wall [26]. Ferulic acid promotes destabilization of the internal fungal cell structure as well as cell surface morphology, induces leakage of intracellular contents, and inhibition of spore germination [27]. Salicylic acid causes leakage of protein content from the cell interior, intracellular disorganization, and lipid damage [28]. Rutin intervenes by affecting the enzymes sterol 14-α demethylase, a key enzyme in the biosynthesis of various sterols, and nucleoside diphosphate kinase, essential in the phosphorylation process of nucleoside triphosphate and diphosphate (NTP and NDP, respectively) [29]. *trans*-Cinnamic acid promotes damage to plasma membrane integrity and also stimulates the increase in intracellular reactive oxygen species, and activities of peroxidase and polyphenol oxidase enzymes [30]. Quercetin has demonstrated a synergistic effect in conjunction with other phenolics, altering the composition of the cell membrane through the induction of oxidative stress [31]. Kaempferol alters the synthesis of nucleic acids and proteins and decreases the function of mitochondria as well as causing disruption of the cell membrane [32].

There are many modes of action through which the phenolic compounds present in the aqueous extract of *V. sphaerocephala* and its fractions could be acting on the phytopathogen *B. cinerea*; however, the authors recommend carrying out a subsequent targeted study to determine their specific mechanisms of action.

It is possible that the antifungal activity observed in this research is due to a synergistic effect of the phenolic compounds present in both the aqueous extract and its ethyl acetate fraction from the stem of *V. sphaerocephala* since no clear relationship was observed between the antifungal activity and the phenolic and flavonoid contents or between the antifungal activity and the individual phenolic compounds identified by UPLC–MS analysis in the aqueous extract and its ethyl acetate fraction.

## 3. Materials and Methods

### 3.1. Chemicals and Reagents 

Sodium carbonate (Na_2_CO_3_) (99%, J.T. Baker^®^, Mexico City, Mexico), aluminum chloride (AlCl_3_) (99%, Meyer^®^, Mexico) potassium persulfate (K_2_S_2_O_8_) (99.8%, J.T. Baker^®^, Phillipsburg, NJ, USA), phosphate dibasic sodium (99.9%, Fermont^®^, Monterrey, Mexico), ammonium molybdate (99%, Merck^®^, Mexico City, Mexico), 2,2′-azino-bis-3-ethylbenzothiazolin-6-sulfonic acid (ABTS) (99%, Roche^®^, Mannheim, Germany), potassium ferrocyanide (K_3_Fe(CN)_6_), trichloroacetic acid (C_2_HCl_3_O_2_), ferric chloride (FeCl_3_) were used. Folin–Ciocalteu reagent (99%) and standard gallic acid (≥99%), 2,2-diphenyl-1-picrylhydrazyl (DPPH), ascorbic acid (≥99%), and rutin hydrate (≥95%) (HPLC grade) were provided by Sigma-Aldrich^®^ (St. Louis, MO, USA).

Potato dextrose agar (PDA) medium (provided by Bioxon^®^, Mexico City, Mexico) and Benomyl^®^ 50 WP (Taicang Pesticide Factory, Jiangsu, China) were used. Water, acetonitrile, and formic acid (LC-MS grade) were purchased from Sigma-Aldrich^®^ (St. Louis, MO, USA). The reference standards used in the UPLC analysis were as follows: shikimic acid, gallic acid, L-phenylalanine, protocatechuic acid, 4-hydroxybenzoic acid, gentisic acid, 4-hydroxyphenylacetic acid, (–)-epigallocatechin, (+)-catechin, vanillic acid, scopolin, chlorogenic acid, caffeic acid, malvin, kuromanin, procyanidin B2, vanillin, keracyanin, (–)-epicatechin, 4-coumaric acid, mangiferin, umbelliferone, (–)-gallocatechin gallate, scopoletin, ferulic acid, quercetin 3,4-di-O-glucoside, 3-coumaric acid, salicylic acid, sinapic acid, epicatechin gallate, ellagic acid, myricitrin, pelargonidin, quercetin 3-D-galactoside, rutin, *p*-anisic acid, quercetin 3-glucoside, luteolin 7-O-glucoside, malvidin, 2,4-dimethoxy-6-methylbenzoic acid, penta-O-galloyl-B-D-glucose, kaemperol 3-O-glucoside, quercitrin, naringin, myricetin, hesperidin, *trans*-resveratrol, rosmarinic acid, secoisolariciresinol, phloridzin, *trans*-cinnamic acid, psoralen, quercetin, luteolin, angelicin, naringenin, apigenin, matairesinol, kaempferol, hesperetin, podophyllotoxin, methyl cinnamate, nordihydroguaiaretic acid, chrysin, kaempferide, emodin, chrysophanol.

### 3.2. Fungal Strain

The fungi *B. cinerea* was previously isolated and identified by Robles-Yerena et al., 2021 [33]. Briefly, *B. cinerea* was isolated from decaying strawberry fruits and was molecularly identified for confirmation of genus and species (accession number: MW698862). This strain belonged to the strain collection of the Molecular Biology Laboratory of the Universidad de La Ciénega del Estado de Michoacán de Ocampo and was donated by Dr. Pedro Damián Loeza-Lara.

### 3.3. Plant Material

*V. sphaerocephala* specimens were collected in October 2020 at Pajacuarán (20°05′54.8″ N, 102°33′19.2″ W) in the Mexican state of Michoacán. The specimens were authenticated by Biol. Rosa Isabel Fuentes Chávez. Voucher herbarium specimens were deposited at the herbarium of the Faculty of Biology of the Universidad Michoacana de San Nicolás de Hidalgo, with herbarium number 31383.

### 3.4. Drying Process

*V. sphaerocephala* plants were dried at room temperature (20 °C) for 5 days in the absence of light. Stems were removed from the plants, and afterward, the tissues were ground in a blender to obtain a fine powder. Milled tissues were stored in dark conditions at room temperature until they were used. 

### 3.5. Preparation of the Extracts

The extract was obtained by maceration in a proportion of 100 g of ground dry tissue (stem) per 500 mL of water at 90 °C, with vigorous shaking every 24 h, in the absence of light for five days [34]. The extract was filtered at room temperature using a Buchner funnel and Whatman No. 1 filter paper (Whatman International Ltd., Maidstone, UK). Then, the extract was evaporated to dryness using a vacuum rotary evaporator (Büchi Waterbath B-480, Flawil, Switzerland) at 50 °C with a rotational speed of 120 rpm for 25 h. Finally, dry extract was suspended in methanol to obtain a final concentration of 100 mg/mL. The extract was stored in amber bottles at 4 °C for later use.

### 3.6. Liquid-Liquid Phase Separation

A liquid-liquid phase separation was carried out on the extract to obtain the fractions [35]. The phases were separated using a separatory funnel, placing 150 mL of extract and 150 mL of solvent (ethyl acetate or methanol). The funnel was shaken well, and phase separation was allowed to occur. The upper organic layer was collected in a beaker. The procedure was repeated two more times. Subsequently, fractions were dried in a rotary evaporator (Büchi Waterbath B-480, Flawil, Switzerland) at 50 °C with a rotational speed of 120 rpm for 8 min and suspended in methanol at a concentration of 100 mg/mL. The fractions were stored at −4 °C.

### 3.7. Determination of Total Phenolic and Flavonoid Content

#### 3.7.1. Total Phenolic Content

Total phenolic content was determined using the Folin–Ciocalteu method, according to Singleton et al. [36], with modifications. Next, 50 µL of each extract and fraction solution was taken, and 500 µL of deionized water was added and mixed. Subsequently, 25 μL of 1 N Folin–Ciocalteu reagent was added and shaken for 5 min. Finally, 75 µL of a 20% (*w/v*) Na_2_CO_3_ solution was added and left to rest in the absence of light for 2 h at room temperature. The absorbances of the samples were read in a Multiskan FC microplate reader (Thermo Fisher Scientific, Waltham, MA, USA) at 760 nm. To determine the phenolic compounds in the aqueous extract and fractions of *V. sphaerocephala*, 200 µL of the sample was taken and evaluated using a gallic acid standard curve (A_760_ = 0.0017 [gallic acid] − 0.0246, *r* = 0.9908), obtained using 10 known concentrations (30–300 μg/mL) of the compound. The total phenolic content was expressed in milligrams of gallic acid equivalents per gram of dry extract (mg GAE/g DE).

#### 3.7.2. Total Flavonoid Content

To determine the total flavonoid content, the technique described by Lamaison and Carnart [37] was used. To achieve this, 250 µL of aqueous extracts and fractions were added with 250 mL of 5% aluminum chloride (*w*/*v*) solution. The absorbance value was evaluated after 10 min at a wavelength of 430 nm in a Multiskan FC microplate reader (Thermo Fisher Scientific, Waltham, MA, USA). The total flavonoid content was determined using a rutin curve (A_430_ = 0.005 [rutin]-0.0062, *r* = 0.9983) obtained using ten concentrations of rutin (0.01–0.1 mg/mL). Total flavonoids were expressed in milligrams of rutin equivalents per gram of dry extract (mg RE/g DE).

### 3.8. Identification and Quantification of Total Phenolic Compounds by Ultra-High-Performance Liquid Chromatography Coupled to Mass Spectrometry

The identification and quantification of phenolic compounds was carried out by the method previously reported [38,39]. The extracts were prepared with the solvent methanol with 0.1% formic acid (*v/v*) at 50 mg/mL, then filtered with 0.2 μm polytetrafluoroethylene (PTFE) membranes and finally placed in 2 mL UPLC vials. All samples were analyzed on a 1290 Infinity Agilent ultra-high resolution liquid chromatograph coupled to a 6460 Agilent triple quadrupole-mass spectrometer (Agilent Technologies, Santa Clara, CA, USA). Chromatography was performed on an Agilent Eclipse Plus C18 column (1.8 µm, 2.1 × 50 mm) with a column temperature of 40 °C. The mobile phase consisted of (A) water (MS grade) and (B) acetonitrile (MS grade), both with 0.1% formic acid (*v*/*v*). The gradient conditions of the mobile phases were 0–30 min a linear gradient 1–50% B, 30–35 min a linear gradient 50–99% B, 35–39 min an isocratic step at 99% B, 39–40 min linear gradient 99–1% B, 40–45 min an isocratic step at 1% B. The flow rate was 0.3 mL/min, and 2 µL of extract was injected. The mass spectrometric analysis was performed with an electrospray ionization source in negative and positive mode with a capillary and nozzle voltages of 3500 and 500 V, respectively. The gas and sheath gas temperatures were 300 and 250 °C, respectively. The gas and sheath gas flows were 5 and 11 L/min, respectively, and the nebulizer pressure was 45 Psi. Data were acquired and processed with the MassHunter workstation software, version B.06.00 (Agilent Technologies, Santa Clara, CA, USA). For identification and quantification, 67 authentic standards were purchased or in-house purified, including two phenolic precursors (shikimic acid and phenylalanine) and 65 phenolic compounds belonging to several subcategories (Supplementary Table S1). The identification of each compound was performed by a dynamic multiple-reaction monitoring method (see detailed information in Supplementary Table S1). The compound quantification was carried out by constructing calibration curves with 12 concentration points from 0.25 to 19 µM, obtaining determination coefficient values higher than 0.96 (Supplementary Table S1). The concentration of each phenolic compound was expressed as micrograms per gram of dry extract (µg/g DE).

### 3.9. Analysis of Antioxidant Activity

#### 3.9.1. DPPH Antioxidant Assay

The DPPH technique was used to determine the antioxidant activity [40]. A DPPH solution was prepared (98 μg/mL in methanol). Then, 900 μL of DPPH reagent was taken to mix with 100 μL of extracts and fractions (5–50 mg/mL), and they were incubated at room temperature for 10 min. The absorbance was read at 523 nm in a Multiskan FC microplate reader (Thermo Fisher Scientific, Waltham, MA, USA). Methanol was used as a blank. The scavenging effect of DPPH was measured using the following formula:

DPPH scavenging effect (%) = [A_control_ − A_sample_ /A_control_] × 100 (1)

where A_control_ is the absorbance of the control (DPPH solution), and A_sample_ is the absorbance of the sample (DPPH plus 100 μL of extract). The median inhibitory concentration (IC_50_) was determined using linear regression. The scavenging activity was expressed as the IC_50_ that represents the *V. sphaerocephala* aqueous extract or fractions concentration (mg/mL) needed to reduce by 50% the initial DPPH absorbance. 

#### 3.9.2. ABTS Antioxidant Assay

The ABTS technique was performed using the procedure described by [41]. The ABTS radical was obtained through the oxidation reaction using 1 mL of the ABTS reagent at a concentration of 7 mmol/L and 17.6 μL of potassium persulfate solution (140 mmol/L). The solution was placed in the absence of light for a period of 12 h at room temperature. The ABTS solution was diluted with water to have an absorbance value of 0.700 ± 0.01 at a wavelength of 734 nm in a Multiskan FC microplate reader (Thermo Fisher Scientific, Waltham, MA, USA). Once the radical was formed, a mixture was made by taking 250 μL of the extracts with 250 μL of ABTS solution. The absorbance value was measured at 734 nm after 6 min. The test was performed with 10 concentrations of extract and fractions (1–50 mg/mL). The blank was prepared under the conditions described above, replacing the aqueous extract with methanol and a fraction with ethyl acetate or methanol as applicable. The percentage of scavenging effect was measured using the following formula:

ABTS scavenging effect (%) = [A_blank_ − A_sample_/A_control_] × 100 (2)

where A_blank_ represents the absorbance of the blank (ABTS solution plus methanol), A_sample_ means the absorbance of the test sample (ABTS solution plus extract), and A_control_ is the absorbance of the control (ABTS solution). The median inhibitory concentration (IC_50_) was determined using linear regression by making a graph with the percentage of inhibition against the concentration of the extract. The scavenging activity was expressed as the IC_50_ that represents the aqueous extract or fractions concentration (mg/mL) needed to reduce by 50% the initial ABTS absorbance. 

#### 3.9.3. Total Antioxidant Capacity

The total antioxidant capacity was carried out according to the reported by [42]. The working solution was prepared with 0.6 M sulfuric acid, 28 mM sodium phosphate, and 4 mM ammonium molybdate. Then, 50 µL of each *V. sphaerocephala* extract and fraction was combined with 500 µL of a working solution. Subsequently, samples were incubated in a dry bath/block heater from Thermo Fisher Scientific (Waltham, MA, USA) at a temperature of 95 °C for 90 min. The samples were then placed at room temperature, and the absorbance was measured at 695 nm in a Multiskan FC microplate reader (Thermo Fisher Scientific, Waltham, MA, USA). A standard curve of ascorbic acid was generated (A_695_ = 0.0014 [ascorbic acid] + 0.0368, *r* = 0.9981, with ten concentrations of ascorbic acid (0.04–0.4 mg/mL). The total values of antioxidant capacity were expressed in milligrams of equivalents of ascorbic acid per gram of dry extract (mg AAE/g DE).

#### 3.9.4. Ferric Reducing Potential

To determine the ferric-reducing power of *V. sphaerocephala* extracts and fractions, the methodology proposed by [43] was used with some modifications. To achieve this, 100 µL of extracts and fractions of *V. sphaerocephala* were mixed with 100 µL of phosphate buffer (0.2 M, pH 6.6) and 100 µL of 1% potassium ferrocyanide (*w*/*v*). The mixture was incubated at 50 °C for 20 min in a digital dry bath/block heater (Thermo Scientific brand, Massachusetts, USA). Then, 100 µL of 10% trichloroacetic acid (*w*/*v*) and 200 µL of deionized water were added. Finally, 40 µL of 0.1% ferric chloride (*w*/*v*) was placed, and the absorbance at 700 nm was recorded in a Multiskan FC microplate reader (Thermo Scientific, Waltham, MA, USA). The ferric reducing power was calculated from the following calibration curve A_700_ = 0.00006 [gallic acid] + 0.00008, *r* = 0.9962, constructed with six concentrations of gallic acid (0.45–2.7 mg/mL). The results were expressed in mg equivalents of gallic acid per gram of dry extract (mg GAE/g ES).

### 3.10. Antifungal Assay

Antifungal activity was determined with the well diffusion method [44], using Petri dishes with 3.9% PDA medium (*w*/*v*) in which 2.5 mm perforations were made. In each well, 100 µL of the extract at six different concentrations (0.01–0.25 mg/mL) or 100 µL of the fraction at eight different concentrations (5–20 mg/mL) were poured. Subsequently, a fragment of *B. cinerea* mycelium was inoculated into each well and incubated for 5 days at 25 ± 2 °C. The commercial fungicide Benomyl^®^ (10 mg/mL) was used as a positive control and methanol as a negative control. The percentage of inhibition (%) was calculated with the formula outlined by Rutiaga [45]:

Inhibition (%) = [Growth_control_ − Growth_sample_/Growth_control_] × 100 (3)

where Growth_control_ represents the average diameter of fungal growth of the control (fungi), Growth_sample_ means the average diameter of fungal growth of the test sample (fungi plus extract or fraction). The median inhibitory concentration (IC_50_) and the lethal inhibitory concentration (IC_100_) were determined using linear regression by making a graph with the percentage of fungi inhibition against the concentration of the extract or fraction. Antifungal activity was expressed as the IC_50_ that represents the aqueous extract or fractions concentration (mg/mL) needed to reduce by 50% the fungal growth, and as the lethal inhibitory concentration (IC_100_), which represents the concentration of aqueous extract or fractions (mg/mL) needed to completely inhibit fungal growth.

### 3.11. Statistical Analysis

Results were reported as mean ± standard deviation of three independent replicates. An analysis of variance (ANOVA) was used to assess statistical significance. Differences between values with a *p* < 0.05 were considered statistically significant. Tukey’s test was performed for the comparison of means for the corresponding results. Relationships between all determinations were tested using Pearson’s correlation. These analyses were performed with the SPSS software version 29.0 (IBM, Armonk, NY, USA). 

## 4. Conclusions

In the present study, the phenolic composition, antioxidant and antifungal activities of the aqueous stem extract, and its fractions (ethyl acetate and methanol) of *V. sphaerocephala* were analyzed. In this study, new phenolic compounds were identified for this species, contributing to its knowledge of phytochemical composition. Our results indicate that the antioxidant activity shown by the aqueous extract and its fractions could be attributed to the phenolic compounds present in them. Also, the antifungal activity exhibited by the aqueous extract of *V. sphaerocephala* stem and its ethyl acetate fraction was highly dependent on the phenolic composition. The results of this research support the idea that the aqueous extract of *V. sphaerocephala* stems possess great potential for *B. cinerea* management. The modes of action are yet to be elucidated. Therefore, future investigations into the specific mechanisms of action of the phenolics detected in the extracts of the fungus *B. cinerea* are recommended.

## Figures and Tables

**Table 1:** Total phenolic and flavonoid contents, free radical scavenging activity, total antioxidant capacity, ferric reducing power of aqueous extract, and fractions from the stem of *Verbesina sphaerocephala*.

Extract	TPC (mg GAE/g DE)	TFC (mg RE/g DE)	DPPH IC_50_ (mg/mL)	ABTS IC_50_ (mg/mL)	TAC (mg AAE/g DE)	FRP (mg GAE/g DE)
Aqueous stem extract	21.40 ± 0.36 ^a^	11.53 ± 0.07 ^a^	12.36 ± 0.07 ^c^	5.60 ± 0.11 ^a^	20.62 ± 0.09 ^c^	74.76 ± 1.48 ^b^
Ethyl acetate fraction	21.26 ± 0.38 ^a^	3.71 ± 0.03 ^b^	7.36 ± 0.01 ^a^	7.76 ± 0.09 ^b^	40.21 ± 0.41 ^a^	129.57 ± 2.08 ^a^
Methanolic fraction	13.54 ± 0.43 ^b^	2.12 ± 0.07 ^c^	8.48 ± 0.02 ^b^	10.26 ± 0.04 ^c^	31.46 ± 0.6 ^b^	61.48 ± 1.04 ^c^

Abbreviations: TPC, total phenolic content; TFC, total flavonoid content; GAE, gallic acid equivalents; RE, rutin equivalents; DE, dry extract; DPPH, 2,2-diphenyl-1-picrylhydrazyl; IC_50_, median inhibitory concentration; TAC, total antioxidant capacity; FRP, ferric reducing power. Values are expressed as mean ± standard deviation of three repetitions. Values with different letters indicate significant differences (Tukey, *p* < 0.05).

**Table 2:** Phenolic compounds identified by Ultra-High-Performance Liquid Chromatography Coupled to Mass Spectrometry (UPLC–MS) in aqueous extract and fractions from the stem of *Verbesina sphaerocephala*.

R_t_ (min)	Compound	µg/g DE		
		Aqueous Extract	Ethyl Acetate Fraction	Methanolic Fraction
1.4	Gallic acid	ND	ND	4.98 ± 0.04 ^a^
2.5	Protocatechuic acid	93.06 ± 2.56 ^b^	250.54 ± 1.04 ^a^	16.14 ± 0.45 ^c^
3.76	Hydroxybenzoic acid	65.69 ± 1.14 ^b^	135.61 ± 0.89 ^a^	3.82 ± 0.12 ^c^
5.12	Vanillic acid	66.86 ± 0.13 ^b^	246.94 ± 3.46 ^a^	5.99 ± 0.26 ^c^
5.34	Chlorogenic acid	22.51 ± 0.34 ^c^	114.66 ± 3.16 ^a^	64.05 ± 1.17 ^b^
5.38	Caffeic acid	ND	18.36 ± 1.16 ^a^	4.86 ± 0.11 ^b^
6.52	Vanillin	10.09 ± 0.06 ^b^	289.27 ± 2.92 ^a^	0.75 ± 0.09 ^c^
7.21	*p*-coumaric acid	21.13 ± 0.45 ^b^	139.72 ± 2.71 ^a^	3.51 ± 0.10 ^c^
8.4	Scopoletin	ND	3.03 ± 0.34 ^a^	ND
8.6	Ferulic acid	2.99 ± 0.15 ^b^	26.05 ± 0.31 ^a^	0.17 ± 0.04 ^c^
9.15	Salicylic acid	10.97 ± 0.25 ^b^	40.19 ± 0.35 ^a^	1.38 ± 0.06 ^c^
9.16	Sinapic acid	1.37 ± 0.21 ^b^	5.74 ± 0.69 ^a^	1.23 ± 0.07 ^b^
10.35	Rutin	20.56 ± 0.73 ^b^	250.52 ± 8.21 ^a^	10.28 ± 0.18 ^b^
10.57	Isoquercitrin (quercetin-3-*O*-glucoside)	ND	69.16 ± 2.19 ^a^	ND
11.91	Astragaline (kaempferol 3-*O*-glucoside)	ND	41.61 ± 1.51 ^a^	ND
13.02	Secoisolariciresinol	ND	47.79 ± 1.09 ^a^	ND
14.08	*trans*-Cinnamic acid	11.46 ± 0.06 ^b^	59.45 ± 1.43 ^a^	ND
15.18	Quercetin	ND	21.25 ± 0.49 ^a^	6.45 ± 0.10 ^b^
15.28	Luteolin	1.07 ± 0.12 ^a^	ND	ND
17.81	Kaempferol	ND	6.94 ± 0.09 ^a^	ND

Abbreviations: R_t_, retention time; DE, dry extract; ND, not determined. Values are expressed as mean ± standard deviation of three repetitions. Values with different letters indicate significant differences (Tukey, *p* < 0.05).

## Data Availability

The authors declare that the data supporting the findings of this study are available within the article.

## Supplementary Materials

The following supporting information can be downloaded at: https://www.mdpi.com/article/10.3390/plants13192791/s1, Figure S1. Representative chromatograms of standards, aqueous extract, ethyl acetate and methanolic fractions from stem of *Verbesina sphaerocephala* compounds identified by Ultra–High Performance Liquid Chromatography Coupled to Mass Spectrometry (UPLC–MS); Table S1. Conditions for identification and quantification of phenolic compounds by UPLC-MS.
