# Supplementary material for: Antioxidant and Antifungal Activities and Characterization of Phenolic Compounds Using Ultra-High Performance Liquid Chromatography and Mass Spectrometry (UPLC-MS) of Aqueous Extracts and Fractions from Verbesina sphaerocephala Stems
Source: Plants (Basel). 2024 Oct 5;13(19):2791. doi: 10.3390/plants13192791 (PMC11479107; doi:10.3390/plants13192791)
Supplement: Supplementary file 1 [file plants-13-02791-s001.zip › plants-3190441-supplementary.pdf]

Table S1. Conditions for identification and quantification of phenolic compounds by UPLC-MS

Compound	dMRM transition			Mass spectrometric conditions			Quantification conditions		
	Precursor ion	Product ion	Retention time	Collision energy	Fragmentor	Polarity	Quantification range (μM)	Regression type	R^2
Shikimic acid[1]	173.1	111.1	0.49	10	100	Negative	0.5 - 19	Quadratic	0.99
Gallic acid[2]	169.0	125.2	1.4	10	100	Negative	1 - 19	Quadratic	0.99
L-Phenylalanine[3]	166.1	131.0	1.92	10	100	Positive	0.25 - 19	Quadratic	0.99
Protocatechuic acid[1]	153.0	109.1	2.5	10	100	Negative	0.25 - 19	Quadratic	0.99
4-Hydroxybenzoic acid[1]	137.1	92.8	3.76	10	100	Negative	0.25 - 19	Quadratic	0.99
Gentisic acid[1]	153.0	109.0	3.83	10	100	Negative	0.25 - 19	Quadratic	0.99
4-Hydroxyphenylacetic acid[3]	107.1	77.0	4.72	20	140	Positive	0.25 - 19	Quadratic	0.99
(-)-Epigallocatechin[4]	305.1	125.0	4.83	20	140	Negative	1 - 17	Quadratic	0.99
(+)-Catechin[2]	291.0	138.9	5.07	10	100	Positive	0.5 - 19	Quadratic	0.99
Vanillic acid[1]	169.0	93.0	5.12	10	100	Positive	0.25 - 19	Quadratic	0.99
Scopolin[5]	355.1	193.0	5.25	20	100	Positive	0.25 - 19	Quadratic	0.99
Chlorogenic acid[1]	355.1	163.0	5.34	10	100	Positive	0.25 - 19	Quadratic	0.99
Caffeic acid[1]	181.0	163.	5.38	10	100	Positive	0.5 - 19	Quadratic	0.99
Malvin[1]	655.1	331.1	5.82	40	100	Positive	0.5 - 19	Quadratic	0.99
Kuromanin[1]	449.0	286.9	6.34	30	100	Positive	0.5 - 19	Quadratic	0.99
Procyanidin B2[1]	577.1	425.1	6.4	10	100	Negative	1 - 19	Quadratic	0.99
Vanillin[1]	153.0	124.9	6.52	10	100	Positive	0.25 - 19	Quadratic	0.99
Keracyanin[2]	595.2	287.1	6.88	20	100	Positive	0.5 - 19	Quadratic	0.99

(-)-Epicatechin[2]	291.0	138.8	6.96	10	100	Positive	0.5 - 19	Quadratic	0.99
4-Coumaric acid[1]	165.0	147.0	7.21	10	100	Positive	0.25 - 19	Quadratic	0.99
Mangiferin[2]	423.0	302.8	7.32	10	100	Positive	0.5 - 19	Quadratic	0.99
Umbelliferone[1]	163.0	107.0	7.64	30	100	Positive	0.25 - 19	Quadratic	0.99
(-)-Gallocatechin gallate[2]	458.9	139.0	7.95	20	80	Positive	1 - 19	Quadratic	0.99
Scopoletin[1]	193.0	133.0	8.4	10	100	Positive	0.25 - 19	Quadratic	0.99
Ferulic acid[1]	195.1	145.0	8.6	20	100	Positive	0.25 - 19	Quadratic	0.99
Quercetin 3,4-di-O-glucoside[1]	627.0	302.9	8.77	10	100	Positive	0.5 - 19	Quadratic	0.99
3-Coumaric acid[1]	165.05	147.04	8.81	10	100	Positive	0.5 - 19	Quadratic	0.99
Salicylic acid[2]	137.0	93	9.15	10	100	Negative	0.5 - 19	Quadratic	0.99
Sinapic acid[1]	225.1	207.1	9.16	10	100	Positive	0.25 - 19	Quadratic	0.99
Epicatechin gallate[4]	443.1	123.0	9.83	10	100	Positive	1 - 19	Quadratic	0.99
Ellagic acid[1]	300.5	145.0	9.98	30	170	Negative	1 - 19	Quadratic	0.99
Myricitrin[1]	465.0	318.9	10.03	10	100	Positive	1 – 19	Quadratic	0.99
Pelargonidin[2]	271.1	121	10.22	20	10	Positive	1 - 19	Quadratic	0.97
Quercetin 3-D-galactoside[2]	465.0	302.9	10.26	10	100	Positive	0.25 - 19	Quadratic	0.99
Rutin[2]	611.0	302.9	10.35	10	100	Positive	0.25 - 19	Quadratic	0.99
p-Anisic acid[3]	153.1	109.0	10.45	5	120	Positive	0.25 - 19	Quadratic	0.99
Quercetin 3-glucoside[2]	465.0	303.0	10.57	10	100	Positive	0.25 - 19	Quadratic	0.99
Luteolin 7-O-glucoside[1]	449.0	287.0	10.77	10	100	Positive	0.5 – 19	Quadratic	0.99
Malvidin[1]	331.1	287.1	11.14	20	100	Positive	1 - 17	Quadratic	0.96

2,4-Dimethoxy-6-methylbenzoic acid[2]	197.0	179.0	11.41	5	80	Positive	0.25 - 19	Quadratic	0.99
Penta-O-galloyl-B-D-glucose[2]	771.1	153.0	11.68	20	100	Positive	0.5 - 19	Quadratic	0.99
Kaemperol 3-O-glucoside[1]	449.0	286.9	11.91	10	100	Positive	0.25 - 19	Quadratic	0.99
Quercitrin[1]	449.1	303.1	11.95	10	100	Positive	0.5 - 19	Quadratic	0.99
Naringin[1]	273.0	153.0	12.13	10	120	Positive	0.25 - 19	Quadratic	0.99
Myricetin[1]	317.0	179.0	12.29	10	100	Negative	0.5 - 15	Quadratic	0.99
Hesperidin[1]	609.1	301.1	12.68	20	100	Negative	0.5 - 19	Quadratic	0.99
trans-Resveratrol[2]	229.1	135.0	12.69	10	100	Positive	0.5 - 19	Quadratic	0.99
Rosmarinic acid[1]	361.1	163.0	12.8	10	100	Positive	0.5 - 19	Quadratic	0.99
Secoisolariciresinol[2]	363.2	137.1	13.02	20	100	Positive	0.5 - 19	Quadratic	0.99
Phloridzin[1]	435.0	272.9	13.04	10	100	Negative	0.25 - 19	Quadratic	0.99
trans-Cinnamic acid[1]	149.1	131.0	14.08	10	100	Positive	0.25 - 19	Quadratic	0.99
Psoralen[1]	187.0	131.1	14.99	20	100	Positive	0.25 - 19	Quadratic	0.99
Quercetin[2]	302.9	153.1	15.18	35	100	Positive	1 - 19	Quadratic	0.99
Luteolin[1]	287.1	153.0	15.28	30	100	Positive	0.5 - 19	Quadratic	0.99
Angelicin[2]	187.0	131.1	15.75	20	100	Positive	0.5 - 19	Quadratic	0.99
Naringenin[1]	271.0	151	16.79	10	100	Negative	0.5 - 19	Quadratic	0.99
Apigenin[1]	271.0	153.0	17.45	30	100	Positive	0.5 - 19	Quadratic	0.99
Matairesinol[4]	359.2	137.1	17.55	10	100	Positive	0.25 - 19	Quadratic	0.99
Kaempferol[1]	287.1	153.0	17.81	30	100	Positive	0.25 - 19	Quadratic	0.99
Hesperetin[1]	303.1	177.1	18.06	20	100	Positive	0.25 - 19	Quadratic	0.99

Podophyllotoxin[4]	415.1	397.1	19.01	10	100	Positive	0.25 - 19	Quadratic	0.99
Methyl cinnamate[2]	163.1	131.0	21.46	6	100	Positive	0.25 - 1	Quadratic	0.99
Nordihydroguaiaretic acid[1]	303.0	193.1	22.72	10	100	Positive	0.5 - 19	Quadratic	0.99
Chrysin[1]	255.1	153.0	22.89	40	100	Positive	0.25 - 19	Quadratic	0.99
Kaempferide[1]	301.0	258.2	24.38	20	100	Positive	0.5 - 19	Quadratic	0.99
Emodin[1]	269.0	225.0	27.45	20	150	Negative	1 - 17	Quadratic	0.99
Chrysophanol[1]	255.1	153.0	31.34	40	100	Positive	0.25 - 19	Quadratic	0.99

The retention time variation allowed for the search of the compounds were 2 min in each case. The cell accelerator voltage was 7 V for each compound. Dilutions were made if the concentration of some compounds were higher than the linearity range. Compounds were purchased from [1]Extrasynthese (Lyon, France), [2]Sigma Aldrich (St. Louis, USA), [3]kindly donated by Dr. Thor Arnason (University of Ottawa), [4]Cayman Chemical Company (Michigan, USA), [5]isolated in-house.

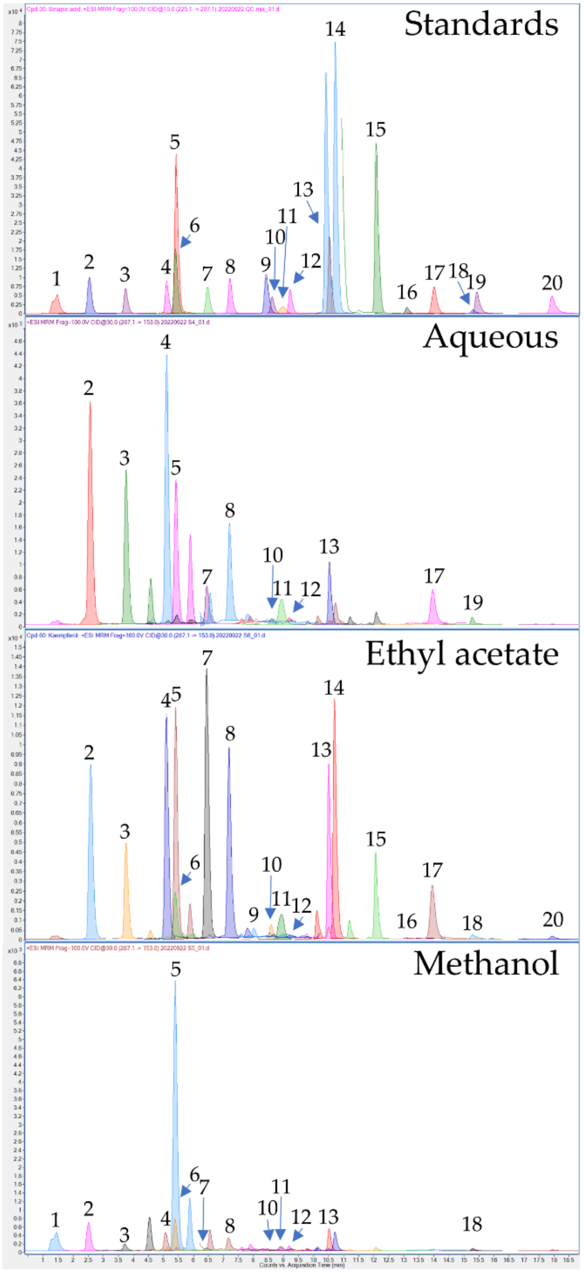

Standards
Aqueous
Ethyl acetate
Methanol

Figure S1. Representative chromatograms of standards, aqueous extract, ethyl acetate and methanolic fractions from stem of *Verbesina sphaerocephala* compounds identified by Ultra–High Performance Liquid Chromatography Coupled to Mass Spectrometry (UPLC–MS). The identified compounds are gallic acid (1), protocatechuic acid (2), 4-hydroxybenzoic acid (3), vanillic acid (4), chlorogenic acid (5), caffeic acid (6), vanillin (7), p-coumaric acid (8), scopoletin (9), ferulic acid (10), salicylic acid (11), sinapic acid (12), rutin (13), Isoquercitrin (14), astragaline (15), secoisolariciresinol (16), trans-cinnamic acid (17), quercetin (18), luteolin (19) and kaempferol (20).
